# Thermal Limits of the Estuarine Amphipod *Melita palmata* Under Different Salinities and Its Relevance for Aquaculture Production

**DOI:** 10.3390/ani16010004

**Published:** 2025-12-19

**Authors:** Luísa Marques, Daniela P. Rodrigues, Rafael C. Duarte, Ricardo Calado

**Affiliations:** ECOMARE, Centre for Environmental and Marine Studies (CESAM), Department of Biology, University of Aveiro, Santiago University Campus, 3810-193 Aveiro, Portugal; dmprodrigues@ua.pt (D.P.R.); rafael.duarte@ua.pt (R.C.D.)

**Keywords:** CTmax, osmoregulation, sustainable aquaculture, feed resource, crustacea, thermal safety margins

## Abstract

Estuarine organisms experience frequent fluctuations in salinity and temperature, facing major challenges to their physiological homeostasis. Such variability can promote high energetic costs for osmoregulation, potentially reducing tolerance to additional stressors. We investigated the effect of salinity on the thermal tolerance of the estuarine amphipod *Melita palmata* (Montagu, 1804), a species of growing interest for aquaculture, either as live feed or as a potential source for essential fatty acids in formulated diets. The critical thermal maximum (CTmax) was determined for males and females collected from three sites within a temperate coastal lagoon (Ria de Aveiro, Portugal) characterized by different salinity regimes (15, 20, and 30). Individuals from lower-salinity environments exhibited significantly lower CTmax values than those from higher salinities, indicating that osmoregulatory costs may restrict thermal resistance. No significant sex-based differences in CTmax were detected. However, thermal safety margins (TSMs) increased with salinity, indicating greater thermal tolerance under higher salinity conditions, and differences in body condition index (BCI) between sites suggest salinity-related effects on growth performance. These results highlight that the elevated energetic demands of osmoregulation under hypo-osmotic conditions can constrain the thermal limits of *M. palmata*, underscoring the complex trade-offs between environmental variability and physiological performance in estuarine habitats. Beyond its ecological implications, understanding the physiological responses of *M. palmata* to salinity and temperature is key, optimising its use in aquaculture. The species’ physiological plasticity under such variable conditions reinforces its suitability for aquaculture production, particularly in earthen ponds in estuarine environments.

## Simple Summary

This study examined how salinity influences the heat tolerance of the estuarine amphipod *Melita palmata*. Individuals were collected from three sites in a coastal lagoon with different salinity levels. Results showed that animals from lower salinity environments were less able to tolerate high temperatures, likely because they spent more energy maintaining their internal balance. In contrast, those from higher salinity sites showed greater heat tolerance and better body condition, suggesting improved overall performance. No significant differences were found between males and females from all three locations. These results indicate that low salinity can reduce the species’ ability to withstand thermal stress. Understanding how *M. palmata* responds to changing salinity and temperature helps explain how estuarine species may cope with environmental change and highlights its suitability for aquaculture production, namely when using earthen ponds in estuarine environments.

## 1. Introduction

Human activities are intensifying pressures on marine ecosystems, leading to substantial ecological consequences, including differences in species distribution and shifts in population dynamics [1,2]. Considering individual-level impacts, marine organisms may be exposed to a range of environmental stressors, including rising sea temperatures [3], ocean acidification driven by decreasing pH [4], increased chemical pollution [5], altered salinity due to freshwater input [6], and declining oxygen availability [7].

In estuarine environments, where salinity naturally fluctuates due to tidal cycles and freshwater inflows [8], osmotic stress poses a significant physiological challenge for resident organisms, particularly those with limited osmoregulatory capacity. Marine invertebrates may undergo enhanced osmotic gradients across body surfaces in response to salinity variations, resulting in passive water influx and ion loss [9,10]. To counteract these osmotic challenges, estuarine organisms possess several physiological mechanisms for osmoregulation, which are metabolically costly to maintain [11,12]. Consequently, these energetic costs may impair the capacity of coastal and estuarine organisms to cope with additional environmental stressors, such as rising temperatures [13,14,15]. Beyond their ecological relevance, these processes are also important in practical settings. Many commercially important aquatic species, particularly crustaceans cultivated in open or semi-open aquaculture systems, are normally exposed to salinity fluctuations similar to those commonly recorded in estuaries. Such variability can affect growth, survival, and energy allocation, making tolerance to osmotic stress a key trait in aquaculture [16,17]. Understanding how environmental variability affects the physiological performance of estuarine organisms is therefore essential, not only for ecological studies but also to diversify and support a more resilient aquaculture.

Thermal tolerance is a key ecological trait that defines a species’ capacity to maintain optimal physiological performance across a range of temperatures [18]. When environmental temperatures exceed this range, physiological function may become impaired, often leading to increased mortality [19]. Species thermal tolerance can vary among populations exposed to different environmental conditions, as well as according to the sex and size of individuals, due to their distinct metabolic demands [20,21]. The critical thermal maximum (CTmax) is a common experimental method used to estimate the upper thermal limit at which an organism’s locomotory activity becomes disorganised, leading to motor coordination failure, loss of response, or muscle spasms, as temperature gradually increases [22,23]. CTmax is widely adopted due to its experimental efficiency, requiring small sample sizes and short trial durations [24,25]. Among ectothermic vertebrates and invertebrates, CTmax is considered a reliable indicator of upper thermal tolerance and provides valuable insight into how species may physiologically respond to environmental changes [26].

Amphipods (Crustacea, Peracarida) are a diverse and ecologically significant group of invertebrates that inhabit environments ranging from freshwater to fully marine ecosystems [27,28]. They are often highly abundant and play key roles in ecosystem functioning, contributing to detritus decomposition, the microbial loop, and nutrient cycling, while also serving as an important trophic link as natural prey for fish and other crustaceans [29,30]. Recently, amphipods have gained attention as a potential sustainable feed for aquaculture due to their unique biochemical composition [31]. They contain high levels of protein, essential amino acids, and omega-3 polyunsaturated fatty acids [32,33,34]. Their tolerance to a wide range of environmental stressors, particularly fluctuations in water salinity, along with traits such as foraging plasticity, migratory behaviour, and drift capacity, has facilitated their successful colonization of diverse habitats worldwide [35,36].

Amphipods exhibit a broad spectrum of ecological affinities; while a considerable number of species are strictly freshwater, many others inhabit brackish, estuarine, or fully marine environments, and some are even adapted to deep-sea conditions [27,28]. Euryhaline species often display greater physiological robustness, as they are exposed to abiotic conditions varying over short and seasonal timescales. For instance, *Gammarus lacustris* populations from saline-lake habitats tolerate thermal and osmotic stress better than conspecifics from freshwater [37]. Similar patterns occur within the genus *Gammarus*, which includes several marine and estuarine species capable of maintaining internal osmotic balance across broad salinity ranges, reflecting strong physiological plasticity in fluctuating brackish and coastal environments [38,39,40]. Moreover, broad salinity tolerance appears associated with ecological plasticity and invasive potential in amphipods, underlining their evolutionary and ecological significance [41]. Given these interspecific differences in physiological and ecological plasticity, using an estuarine or marine amphipod species with broad salinity tolerance should be prioritized for aquaculture settings to achieve a stable biomass production, high survival, and maintenance of nutritional quality under variable conditions [32,33,34,41]

The estuarine amphipod *Melita palmata*, Montagu, 1804 (Figure S1) is a common species in temperate coastal lagoons, estuaries, and brackish waters throughout the North Atlantic, Mediterranean, and Black Sea regions [42,43,44]. It has also been reported as an exotic species in estuarine habitats across the Southwestern Atlantic [45,46]. Individuals of this species are laterally compressed and sexually dimorphic, with males typically being larger than females and possessing enlarged gnathopods. Adult body size varies with environmental conditions, but it generally ranges from 5 to 14 mm [43,47]. Due to its sensitivity to changes in salinity, pollution, and temperature, *M. palmata* is considered a valuable bioindicator for environmental stress [48,49]. As other amphipods, tolerance to salinity changes is linked to active ionic regulation, including increased gill Na^+^/K^+^-ATPase activity [50], adjustments in ion and water fluxes [51], and the involvement of gill ionocytes in Na^+^ and Cl^−^ uptake [52]. At the same time, the physiological plasticity of *M. palmata* facilitates its use for aquaculture production, making it possible to achieve stable biomass production and maintain high nutritional quality. Such resilience is particularly advantageous in systems under daily or seasonal fluctuations in salinity and temperature, as well as in those promoting the reuse of brackish or marine water to achieve more sustainable production. These traits make *M. palmata* a promising candidate for cultivation in open or semi-open earthen pond systems [32,34,53,54,55]. Furthermore, its rapid growth, detritivorous feeding habits, and ability to utilize organic matter as food enhance its value as a low-trophic feeding organism, in line with circular bioeconomy principles.

In this study, we determined the CTmax of male and female *M. palmata* collected from three locations that differ in overall salinity conditions (15, 20, and 30) within a temperate coastal lagoon (Ria de Aveiro, Portugal). We predicted that individuals from habitats experiencing greater abiotic variability, particularly more pronounced salinity fluctuations, would exhibit lower thermal limits (i.e., lower CTmax), as increased energetic investment in osmoregulation may constrain their capacity to cope with thermal stress. Sex-related differences in CTmax were also anticipated; females and males may differ in thermal limits due to reproductive energetic demands [20,56] and body size, given that thermal tolerance in crustaceans often decreases with increasing size [56,57]. We further predicted that individuals of *M. palmata* would be lighter for a given size in habitats with greater salinity variations, since elevated energetic costs for osmoregulation could limit growth and resource allocation [58]. Alternatively, from an adaptive perspective, populations exposed to stronger environmental fluctuations may evolve larger body sizes as a compensatory strategy, enhancing energy storage and fecundity [59]. Understanding these physiological responses is essential for assessing the adaptive potential of *M. palmata* to variable aquaculture environments and optimising its production.

## 2. Materials and Methods

### 2.1. Ethical Statement

Amphipod crustaceans are not currently framed under EU legislation on animal experimentation (European Directive 2010/63/EU, and their corresponding Portuguese legislation, Decree-Law 113/2013). Nevertheless, all the performed experimental procedures complied with these regulations and the three Rs principles of animal welfare. The authors L.M. and D.P.R. hold a B-level certification for animal experimentation in compliance with FELASA (Federation of European Laboratory Animal Science Association) (license number 0421/000/000, registered by the Portuguese National Authority for Food and Animal Health).

### 2.2. Sampling Sites and Amphipod Collection

**Figure 1:**
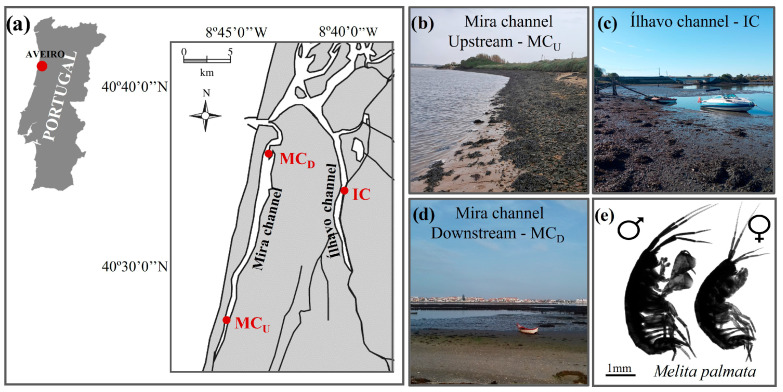
Map (**a**) and photographs (**b**–**d**) showing the location and general characteristics of the sampling sites Mira channel—upstream (MC_U_) (**b**), Ílhavo channel (IC) (**c**), and Mira channel—downstream (MC_D_) (**d**) spanning a natural salinity gradient within Ria de Aveiro coastal lagoon (Aveiro, Portugal), where male and female individuals of the estuarine amphipod *Melita palmata* (**e**) were collected.

Specimens of *M. palmata* were collected during low tide in January 2025 from three distinct sites within Ria de Aveiro coastal lagoon (Portugal) (Figure 1a). January was chosen because it represents one of the coldest periods of the year, when organisms are likely acclimatised to the lowest ambient thermal conditions. Under these conditions, organisms are particularly suitable for upper-thermal-limit trials, as using winter-collected individuals will maximize the contrast between their acclimation baseline and the elevated temperatures applied during the experiment, thereby enhancing the ecological and physiological relevance of the results. This approach aligns with evidence showing that, in aquatic ectotherms, thermal tolerance is strongly shaped by recent thermal history and acclimation temperature [60]. Seasonal plasticity has also been widely reported, where organisms collected during colder periods tend to exhibit lower baseline temperatures and broader thermal safety margins, which makes their responses to heat-stress trials more pronounced and enhances the realism of assessments under extreme warming scenarios [60,61]. By performing experiments on winter-collected organisms, we minimize the effects of prior warm acclimation and better simulate extreme warming events, providing a more accurate estimate of species’ upper thermal limits and vulnerability to climate-induced thermal stress.

The first site is located in the upstream section of Mira channel, approximately 13 km from the inlet connecting this coastal lagoon to the Atlantic Ocean (MC_U_; 40°33′03.5″ N, 8°46′09.4″ W) (Figure 1b). This area is characterized by mixed sandy-muddy sediments and strong freshwater inputs from nearby rivers and ponds, resulting in pronounced tidal and seasonal salinity fluctuations, typically ranging from 10 to 15 [62,63]. The second site, located in Ílhavo channel (IC; 40°34′31.9″ N, 8°40′47.9″ W) (Figure 1c), comprises extensive intertidal mudflats where the mixing of marine and freshwater inputs produces brackish conditions, with salinity values between 15 and 20 [62,63,64]. Finally, the third site is located about 3 km from the ocean entrance, in the downstream section of Mira channel (MC_D_; 40°37′07.8″ N, 8°44′24.2″ W). This area features fine sandy–muddy substrates and is strongly influenced by marine conditions, with salinities typically ranging from 30 to 35 and limited tidal and seasonal variability in environmental parameters [62,63,64,65,66] (Figure 1d). Additional details on annual water temperatures (mean ± SD) across seasons and tidal stages for the three sampling sites are presented in Table S1. At each site, *M. palmata* specimens were collected by gently shaking fronds of various macroalgal species (e.g., *Ulva* sp., *Gracilaria* sp., *Fucus* sp., among others) inside a 20-L bucket with a 1-mm mesh bottom. The collected organisms were transported in local water to the nearby laboratory (CEPAM-ECOMARE, University of Aveiro, Portugal). Salinity and temperature were measured in situ, with three replicate measures being recorded for each parameter at each sampling site (Table S2).

### 2.3. Critical Thermal Maximum Reference and Body Size of Melita palmata

Sampled specimens of *M. palmata* were first sorted by sex based on the presence of enlarged gnathopods in males. Individuals were then acclimated in the laboratory for 24 h in three 6-L tanks containing synthetic seawater prepared by mixing purified tap water (using reverse osmosis) with Red Sea^®^ Coral Pro salt, following the manufacturer’s instructions. The 24-h acclimation period was considered appropriate to allow individuals to recover from handling and transport and to verify that only healthy specimens were used in the CTmax trial. This procedure ensures that any observed responses during the CTmax trial reflect natural thermal limits rather than transient effects from collection or transport. Synthetic water was used to maintain a constant and reproducible salinity throughout the trial [67,68,69], ensuring that individuals from each sampling site were kept at the salinity corresponding to their site of origin. Tanks were maintained at 15 °C (i.e., the mean temperature recorded on the sampling day), pH 8, with continuous aeration and under a 12 h light:12 h dark photoperiod. Salinity levels during acclimation were adjusted to match the site-specific averages reported for each location [64,70], set at 15 for MC_U_, 20 for IC, and 30 for MC_D_. The critical thermal maximum (CTmax) of each individual was assessed following the methodology described by Cuculescu et al. [71] and adapted by Madeira et al. [18]. Ten males and ten females from each sampling site were individually placed in 250-mL glass beakers filled with synthetic seawater adjusted to the salinity corresponding to their original site, with each individual representing a replicate. Each beaker was individually aerated and randomly placed in a thermal bath initially set to 15 °C. The thermal bath consisted of a 260-L tank filled with freshwater and equipped [68] with a digitally controlled heater (JUMO, Quantrol PID LC300, JUMO GmbH & Co. KG, Fulda, Germany, with a temperature probe PT1000 coupled to an aluminium box IP66 and a 3 kW heater, Aqualgae Soc. Lda). Water temperature from the thermal bath was then gradually increased at a constant rate of 2 °C per hour, starting from the initial environmental temperature (15 °C), until all organisms reached their CTmax. The endpoint (Tendpoint) was defined as the temperature at which an individual no longer maintained equilibrium, exhibiting disorganized or ineffective movements and failing to right itself when gently disturbed, marking its critical thermal threshold, with water temperature at this specific moment being recorded. To ensure that the CTmax protocol did not induce behavioural changes unrelated to temperature stress in the amphipods, a negative control trial was also performed. The same experimental design (ten replicates per sex and site) was used under identical conditions, except that temperature was maintained stable at 15 °C throughout the same time frame as conspecifics exposed to increasing water temperature. Amphipods remained in the system for approximately nine hours, corresponding to the average duration of CTmax trials.

After CTmax assessment, each specimen was photographed under a stereomicroscope, and its metasomatic length (defined as the distance between the anterior end of the rostrum and the posterior end of the last metasomatic segment, in mm) was measured using the ImageJ 1.53e software. This metric was then used to indirectly estimate the total length (TL) of each specimen through a standard equation [72]. Amphipods were subsequently frozen at −18 °C and freeze-dried (Labogene Scanvac Coolsafe) for dry weight (DW) determination using a precision balance (Sartorius Competence CPA225D, d = 0.01 mg).

Thermal safety margins (TSMs) are common metrics evaluated in ecophysiological studies and are normally estimated by subtracting the maximum habitat temperature (MHT) recorded in the field from the mean estimated CTmax. However, because single or even seasonal field measurements may not accurately capture long-term thermal variability, we used temperature data series reported in previous studies [70] to obtain more reliable estimates of MHT for each location (MC_U_, IC, and MC_D_). Specifically, the mean summer temperature [70] was used as a proxy for MHT, yielding MHT_mean_ values of 25 °C at MC_U_, 25 °C at IC, and 22 °C at MC_D_. These estimates closely matched previously reported data from field surveys and modelling studies [64,70,73,74,75].

### 2.4. Statistical Analyses

All statistical analyses were performed using the software R v.4.4.3 [76]. A two-way Analysis of Covariance (ANCOVA) was performed to evaluate the effects of site-specific salinity level (15, 20, or 30) and sex on the CTmax of *M. palmata* amphipods, while controlling for individual DW. DW was used as a covariate instead of TL due to a strong positive correlation between the two variables (Pearson’s correlation: r = 0.776; t_58_ = 9.38; *p* < 0.001) and because TL was indirectly estimated from metasomatic length, which could introduce involuntary bias. We also calculated a body condition index (BCI) to examine whether individuals of *M. palmata* from different sampling sites and exposed to site-specific salinity conditions differ in weight from a linear regression model fitting the relationship between log-transformed DW and TL. This approach is a standard, well-established index, widely used in ecological research, and has demonstrated a reliable performance in multiple validation studies [77,78]. To account for possible differences between sexes, we first tested a model including the interaction between sex and TL. Since the interaction was not significant (F_1,56_ = 0.06; *p* = 0.807), sex was retained only as a covariate, reflecting the sexual dimorphism in the species (males generally being larger and heavier than females). In this framework, positive residuals indicate individuals heavier than expected for their size. Differences in the BCI among sites were then evaluated with a one-way ANOVA. For all statistical tests, the assumptions of normality of residuals and the homogeneity of variances were visually assessed through q-q plots and the Bartlett test, respectively, using the “performance” package in R [79]. When significant effects were detected, Tukey’s post-hoc tests were applied to identify differences between factor levels, using the “emmeans” package [80].

## 3. Results

All amphipods used in the CTmax experiment were healthy and active adults, as confirmed after the 24-h acclimation period. Ovigerous females were intentionally excluded to avoid behavioural and physiological biases associated with brooding. The final dataset consisted of males and non-ovigerous females from all three sampling locations, with the mean TL and DW of each group being summarized in Table S3.

**Figure 2:**
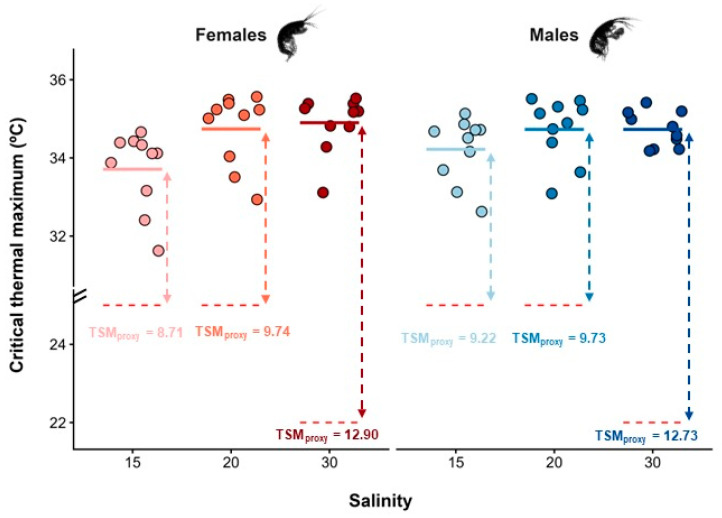
Critical thermal maximum (CTmax, °C) estimated for female and male *Melita palmata* amphipods collected from sampling sites with distinct average salinity levels (15, 20, and 30) in Ria de Aveiro, Portugal. Thermal safety margins (TSMs, °C) were calculated as the difference between the average CTmax of females and males per salinity level and the mean maximum habitat temperature (MHT_mean_, °C) retrieved from the literature of the corresponding site, which are represented by the red dotted lines. Filled circles represent raw data, and the solid-coloured lines represent the mean CTmax for each combination of sex and salinity level.

Amphipods in the control group showed no abnormal behaviour, mortality, or changes in swimming activity, confirming that behavioural shifts recorded in the experimental groups, such as changes in movement patterns and loss of equilibrium, were caused by increasing water temperature. During CTmax trials, amphipods were continuously monitored, with the first observable behavioural change occurring at approximately 24 °C, when one individual exhibited continuous vertical swimming. This behaviour was subsequently observed in multiple individuals at varying temperatures, ranging from 24 °C to 30 °C (Figure 2). As the water temperature rose to approximately 29–30 °C, several amphipods exhibited reduced locomotor activity but remained responsive to gentle external stimulation, applied as a light touch with a spatula to evaluate mobility, with no loss of equilibrium detected. As temperatures continued to increase, reaching 33 °C, some individuals remained near the surface, clinging to the aeration tube, before ultimately experiencing complete loss of motor function. The lowest recorded CTmax values were 31.6 °C for females and 32.6 °C for males, both from the MC_U_ site. In contrast, the highest recorded values were 35.6 °C for females from IC and 35.4 °C for males from MC_D_ (Figure 2).

**Figure 3:**
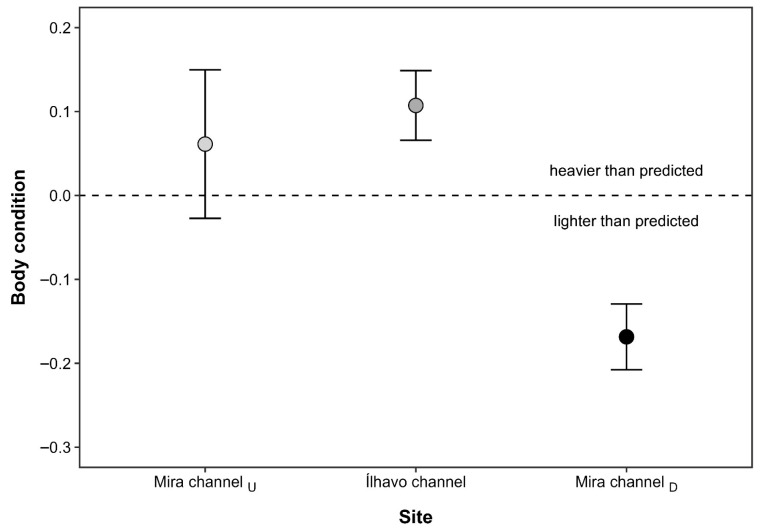
Body condition index (mean ± SE) of *Melita palmata* amphipods collected from three locations within Ria de Aveiro (Portugal): the upstream section of the Mira channel (Mira channel_U_), the Ílhavo channel, and the downstream section of the Mira channel (Mira channel_D_), which differ in average salinity levels. Positive values indicate individuals heavier than predicted for their body length, whereas negative values indicate lighter individuals.

The CTmax values of *M. palmata* were significantly influenced by salinity (F_2,53_ = 6.80; *p* = 0.002), but not by sex (F_1,53_ = 0.31; *p* = 0.579), nor by the interaction between salinity and sex (F_1,53_ = 3.80; *p* = 0.057), after controlling for individual DW. The covariate DW did not significantly affect CTmax (F_1,53_ = 3.80, *p* = 0.057), indicating that thermal limits were independent of individual body mass. Regardless of sex, amphipods from MC_U_ exposed to the lowest salinity level displayed significantly lower CTmax values (mean ± SE: 33.96 ± 0.21 °C) than individuals from both IC (34.73 ± 0.19 °C) and MC_D_ (34.81 ± 0.13 °C), which did not differ from each other (Figure 2). Interindividual differences in CTmax responses were generally higher for females (coefficient of variation: CV = 2.96%) than males (CV = 2.13%), and higher for individuals from MC_U_ (CV = 2.74%) and IC (CV = 2.45%), when compared with those from MC_D_ (CV = 1.72%). The highest TSM was registered at MC_D_ (12.90 °C for females, and 12.73 °C for males), indicating that specimens of *M. palmata* at this location had a higher thermal resistance than those at the other sites. Similarly, the BCI of *M. palmata* also differed significantly across sampling sites (F_2,57_ = 5.90; *p* = 0.005). Individuals from MC_D_ were significantly lighter than expected for their body length when compared to amphipods from both MC_U_ and IC populations, which did not differ from each other (Figure 3).

## 4. Discussion

The present study provides important insights into how natural salinity gradients shape the thermal limits of the estuarine amphipod *M. palmata*, offering a broader understanding of how macroinvertebrates inhabiting dynamic coastal environments respond to interacting environmental stressors. Our findings show that individuals maintained under low salinity conditions, consistent with their habitat conditions, exhibited significantly lower CTmax values compared with conspecifics from higher-salinity sites, suggesting that osmotic stress can reduce thermal resilience. However, such physiological stress did not translate into reduced body condition among individuals from the most salinity-stressful site, indicating potential long-term metabolic adaptation to lower salinity. Our results suggest that the elevated energetic costs of osmoregulation in highly variable estuarine environments may limit the physiological capacity of *M. palmata* to withstand additional thermal stress, although without apparent consequences for growth performance. These findings underscore the importance of accounting for multiple interacting stressors when assessing species’ vulnerability to climate change.

Although our design cannot disentangle short-term plasticity from long-term adaptation, both remain plausible explanations for our findings. Future studies on this topic can be specifically designed to answer whether the observed trend of osmotic stress decreasing thermal resilience is a result of acclimation (plasticity) or local adaptation. Salinity and temperature interact to affect metabolism, ion regulation, and thermal tolerance in estuarine invertebrates. For instance, amphipods from low-salinity habitats have shown lower heat resistance, likely reflecting the energetic costs of osmoregulation under hypoosmotic conditions [37]. At the same time, some crustacean populations exhibit distinct tolerance phenotypes linked to their environmental conditions or thermal regimes, suggesting that population-level divergence may arise under spatially heterogeneous habitats [81,82].

The lower CTmax values observed for *M. palmata* individuals from the MC_U_ site (i.e., the location with the lowest salinity) are consistent with previous findings indicating that estuarine organisms tend to be more sensitive to thermal challenges at lower salinities [14,37]. Recently, a meta-analysis provided strong support for this pattern, demonstrating that low salinity levels consistently increased thermal sensitivity of individuals across a range of taxa, including algae, invertebrates, and fish [14]. As with other crustaceans, amphipods generally function as osmoconformers under high salinity levels, maintaining internal osmotic conditions that are nearly isotonic with the surrounding environment. In contrast, at lower salinities, they switch to a hyperosmoregulatory mode, actively maintaining hemolymph ion concentrations above those of the external medium [10]. Physiological adjustments to changing salinities are energetically demanding and involve mechanisms such as the uptake of ions, particularly sodium and chloride, along with reduced water permeability through the organism’s exoskeleton [10,11,12]. Consistent with these observations, *M. palmata* individuals inhabiting the lowest salinity site are likely under greater osmotic stress, coping with elevated metabolic costs associated with osmoregulation. This increased energetic burden may constrain the energy available to generate an effective response to acute thermal stress, ultimately resulting in reduced thermal limits, as reflected by lower CTmax values. Further research is needed to clarify the physiological mechanisms underlying these patterns and to assess the broader implications of salinity-driven thermal sensitivity for the resilience of estuarine amphipod populations and species under climate change scenarios. Contrary to our initial hypothesis, thermal tolerance (CTmax) did not differ significantly between males and females of *M. palmata* across salinity conditions, consistent with patterns reported for diverse terrestrial and aquatic taxa [56,83]. However, greater female sensitivity to acute thermal stress has already been observed in some amphipod species. These differences are often attributed to sex-specific metabolic demands and energy allocation strategies, with females typically investing more in reproduction at the expense of physiological stress responses. For example, females of the freshwater amphipod species *Gammarus roeseli* showed reduced survival, impaired pleopod ventilation, and disrupted ion regulation under salinity stress compared to males [84]. Similarly, in *Eulimnogammarus verrucosus* from Lake Baikal, females exhibit lower expression of heat shock proteins than males, suggesting increased thermal sensitivity in the first [85]. In the present study, ovigerous females were not included, and sexual size dimorphism in *M. palmata* is relatively small, in comparison to other amphipod species already investigated, which may explain the absence of a detectable sex effect. Additionally, both males and females inhabit the same highly variable estuarine environment, likely experiencing similar selective pressures on thermal tolerance [86]. Interestingly, studies reporting sex-based differences in responses to salinity and temperature stress between sexes have focused primarily on freshwater amphipods. Whether reproductive investment in females increases in species adapted to low-salinity environments and, in turn, affects their responses to physiological stress, compared to those from brackish or marine habitats, remains unclear and warrants further comparative experimental research.

Regardless of sex, *M. palmata* from sites with lower salinity (MC_U_ and IC) experience greater environmental variability, driven by freshwater input and seasonal fluctuations in both temperature and salinity. Such unstable environmental conditions are known to promote acclimation capacity and physiological plasticity [87,88], but they also impose higher energetic demands associated with osmoregulation, which can ultimately constrain the species’ resilience to additional thermal stress. Studies on different *Gammarus* species suggest that the increased energy expenditure required for osmoregulation under hypo-osmotic conditions may reduce the energy available for growth and for coping with other stressors, such as temperature [37,38,39].

Although previous studies have reported that individuals from low-salinity environments often exhibit smaller body sizes and reduced thermal tolerance [58,89], *M. palmata* from the lower-salinity sites in our study showed significantly higher BCI, indicating that specimens from this location were heavier than expected for their body size. This contrast may reflect species-specific osmoregulatory adaptations that mitigate the energetic constraints typically associated with hypo-osmotic stress. In particular, *M. palmata* may possess efficient ion-regulatory or metabolic mechanisms that reduce the energetic burden of maintaining homeostasis at low salinity, thereby allowing greater energy allocation to somatic growth. Additionally, the absence of direct competitors at the MC_U_ and IC sites may enhance resource availability and occupation of macroalgal hosts, promoting an improved growth performance. Finally, higher BCI values could also result from increased food availability or organic enrichment, which may enhance the nutritional condition of individuals inhabiting brackish waters [90]. Future research should investigate how environmental conditions, together with sex-specific physiological responses, influence thermal tolerance, growth rates, and survival strategies in estuarine and coastal amphipod species under changing climatic conditions.

Beyond their ecological relevance, our results also have practical implications for the growing interest in using amphipods as live feeds and sustainable ingredients for aquafeeds [32,34,53,54,55]. Understanding how environmental factors shape amphipod physiological performance is essential for optimizing culture conditions, ensuring stable biomass production, and maintaining nutritional quality under variable salinity and temperature regimes commonly observed in aquaculture systems, particularly in open or semi-open earthen ponds. Amphipods capable of tolerating elevated temperatures, especially under brackish or marine salinities, may have a competitive advantage in such systems, where environmental fluctuations are frequent and intense [14,37]. Therefore, the physiological plasticity and tolerance of estuarine amphipod species such as *M. palmata* to variable salinities and temperatures highlight their potential as promising candidates for aquaculture production, thus helping to diversify the potential sources of high-quality lipids and proteins for the formulation of aquafeeds.

## 5. Conclusions

Our study demonstrates that *M. palmata* amphipods from low-salinity environments exhibit reduced thermal limits compared to conspecific populations from high-salinity sites, indicating that the energetic demands of osmoregulation under hypo-osmotic conditions can constrain their capacity to tolerate higher water temperatures. However, higher BCI values at low salinity suggest long-term physiological adaptation and sustained investment in somatic growth despite osmotic stress. These salinity-dependent differences in thermal limits emphasize the importance of considering multiple, interacting stressors when assessing the resilience of estuarine amphipods under future climate scenarios. Overall, this study advances our understanding of the physiological plasticity capacity and adaptive capacity of estuarine organisms, offering a foundation for predicting their persistence under environmental change. Future research should further examine how environmental variability shapes the physiological performance and resilience of *M. palmata* and other amphipod species. This will help to support their potential use in aquaculture and promote their integration into sustainable production systems. A better understanding of these processes will not only help elucidate species’ adaptive responses in natural environments but also facilitate their use in aquaculture.

## Data Availability

All data generated and analysed for the current study are available on Figshare. https://figshare.com/s/f56e2bf28a9fe183c08a. accessed on 13 November 2025.

## Supplementary Materials

The following supporting information can be downloaded at: https://www.mdpi.com/article/10.3390/ani16010004/s1, Figure S1. Male and female specimens of the amphipod, *Melita palmata*. Table S1. Annual water temperatures (°C) (mean ± SD, n=3) measured at the three sampling sites: Mira channel upstream (MC_U_), Ílhavo channel (IC), and Mira channel downstream (MC_D_), across low, intermediate, and high tides during winter, spring, summer, and autumn. Table S2. Salinity and temperature (mean ± SD, n=3) measured on the sampling day, at the three sampling sites: Mira channel upstream (MC_U_), Ílhavo channel (IC), and Mira channel downstream (MC_D_). Table S3. Dry weight (mg) and total length (TL, in mm) of female and male *Melita palmata* collected at the three sampling sites: Mira channel upstream (MC_U_), Ílhavo channel (IC), and Mira channel downstream (MC_D_) (mean ± SD). Total length was estimated from metasomatic length (ML, in mm), measured from the anterior end of the rostrum to the posterior end of the last metasomatic segment, using a standard conversion equation: TL=-0.153+1.218*ML.
