# Supplementary material for: Thermal Limits of the Estuarine Amphipod Melita palmata Under Different Salinities and Its Relevance for Aquaculture Production
Source: Animals (Basel). 2025 Dec 19;16(1):4. doi: 10.3390/ani16010004 (PMC12785059; doi:10.3390/ani16010004)
Supplement: Supplementary file 1 [file animals-16-00004-s001.zip › animals-4014990-supplementary.pdf]

Supplementary Material

Thermal Limits of the Estuarine Amphipod *Melita palmata* Under Different Salinities and Its Relevance for Aquaculture Production

Luísa Marques *,†, Daniela P. Rodrigues †, Rafael C. Duarte and Ricardo Calado *

ECOMARE, Centre for Environmental and Marine Studies (CESAM), Department of Biology, University of Aveiro, Santiago University Campus, 3810-193 Aveiro, Portugal; dmprodrigues@ua.pt (D.P.R.); rafael.duarte@ua.pt (R.C.D.)

* Correspondence: luisa.marques@ua.pt (L.M.); rjcalado@ua.pt (R.C.)

† These authors contributed equally to this work.

Figure S1. Male and female specimens of the amphipod, *Melita palmata*.

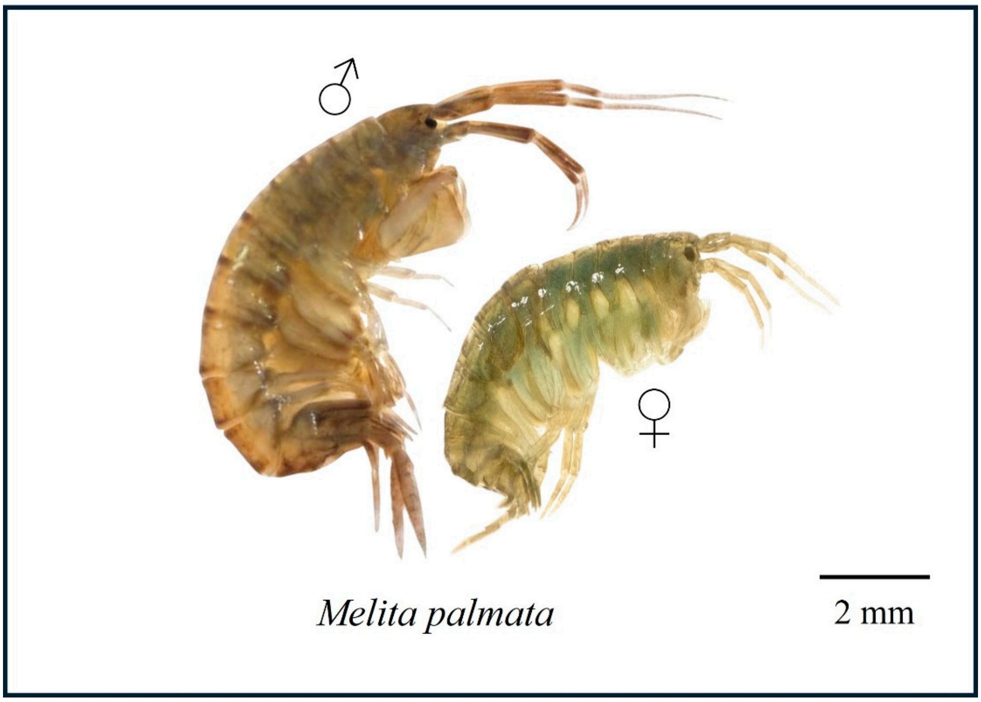

Table S1. Annual water temperatures (°C) (mean ± SD, n=3) measured at the three sampling sites: Mira channel upstream (MC_U), Ílhavo channel (IC), and Mira channel downstream (MCD), across low, intermediate, and high tides during winter, spring, summer, and autumn.

**Season**	**Site**	**Tide**	**Temperature** (°C) **± SD**	**Season**	**Site**	**Tide**	**Temperature** (°C) **± SD**
**winter**	MC_U	low	13.6 ± 0.21	**spring**	MC_U	low	22.8 ± 0.20
		intermediate	15.0 ± 0.50			intermediate	24.1 ± 0.29
		high	15.3 ± 0.17			high	23.4 ± 0.47
	IC	low	13.3 ± 0.15		IC	low	23.4 ± 0.35
		intermediate	17.4 ± 0.75			intermediate	26.2 ± 0.44
		high	15.2 ± 0.15			high	25.0 ± 0.06
	MC_D	low	13.9 ± 0.26		MC_D	low	20.2 ± 0.30
		intermediate	15.7 ± 1.30			intermediate	21.4 ± 0.25
		high	16.0 ± 0.31			high	22.0 ± 0.40
**Season**	**Site**	**Tide**	**Temperature** (°C) **± SD**	**Season**	**Site**	**Tide**	**Temperature** (°C) **± SD**
**summer**	MC_U	low	24.4 ± 0.52	**autumn**	MC_U	low	10.6 ± 0.15
		intermediate	24.9 ± 0.44			intermediate	11.7 ± 0.32
		high	25.6 ± 0.47			high	12.0 ± 0.06
	IC	low	19.7 ± 0.31		IC	low	10.2 ± 0.46
		intermediate	25.5 ± 0.70			intermediate	12.5 ± 1.40
		high	21.2 ± 0.25			high	11.9 ± 0.00
	MC_D	low	17.0 ± 0.35		MC_D	low	12.2 ± 0.35
		intermediate	21.5 ± 1.61			intermediate	13.9 ± 0.38
		high	22.9 ± 0.83			high	14.9 ± 0.20

Table S2. Salinity and temperature (mean ± SD, n=3) measured on the sampling day, at the three sampling sites: Mira channel upstream (MC_U), Ílhavo channel (IC), and Mira channel downstream (MC_D).

	**Salinity**	**Temperature** (°C)
MC_U	8.02 ± 4.29	14.70 ± 0.94
IC	10.78 ± 4.60	15.31 ± 1.79
MC_D	26.69 ± 4.93	15.18 ± 1.18

Table S3. Dry weight (mg) and total length (TL, in mm) of female and male *Melita palmata* collected at the three sampling sites: Mira channel upstream (MC_U), Ílhavo channel (IC), and Mira channel downstream (MC_D) (mean ± SD). Total length was estimated from metasomatic length (ML, in mm), measured from the anterior end of the rostrum to the posterior end of the last metasomatic segment, using a standard conversion equation: $TL = -0.153 + 1.218 * ML$.

	Dry weight (mg)		Total length (mm)	
**Females**	MC_U	2.16 ± 0.93	MC_U	6.33 ± 0.85
	IC	1.54 ± 0.31	IC	4.45 ± 0.59
	MC_D	1.05 ± 0.32	MC_D	3.91 ± 0.78
**Males**	MC_U	4.27 ± 0.67	MC_U	7.63 ± 0.23
	IC	3.27 ± 0.46	IC	6.13 ± 0.97
	MC_D	2.39 ± 0.51	MC_D	5.75 ± 1.29
